# Peripheral neuropathy induced by drinking water contaminated with low-dose arsenic in Myanmar

**DOI:** 10.1186/s12199-019-0781-0

**Published:** 2019-04-23

**Authors:** Hitoshi Mochizuki, Khin Phyu Phyu, Myo Nanda Aung, Phyo Wai Zin, Yasunori Yano, Moe Zaw Myint, Win Min Thit, Yuka Yamamoto, Yoshitaka Hishikawa, Kyaw Zin Thant, Masugi Maruyama, Yoshiki Kuroda

**Affiliations:** 1 Division of Neurology, Respirology, Endocrinology and Metabolism, Department of Internal Medicine, Faculty of Medicine, University of Miyazaki, 5200 Kihara, Kiyotake, Miyazaki, 889-1692 Japan; 2 Department of Medical Research, Ministry of Health and Sports, Yangon, Myanmar; 3 Center for International Relations, University of Miyazaki, Miyazaki, Japan; 4 Department of Neurology, Yangon General Hospital, Yangon, Myanmar; 5 Department of Public Health, Faculty of Medicine, University of Miyazaki, Miyazaki, Japan; 6 Department of Anatomy, Histochemistry and Cell Biology, Faculty of Medicine, University of Miyazaki, Miyazaki, Japan; 7 Department of Applied Physiology, Faculty of Medicine, University of Miyazaki, Miyazaki, Japan

**Keywords:** Arsenic, Pollution, Groundwater, Peripheral neuropathy, Sensory disturbance

## Abstract

**Background:**

More than 140 million people drink arsenic-contaminated groundwater. It is unknown how much arsenic exposure is necessary to cause neurological impairment. Here, we evaluate the relationship between neurological impairments and the arsenic concentration in drinking water (ACDW).

**Participants and methods:**

A cross-sectional study design was employed. We performed medical examinations of 1867 residents in seven villages in the Thabaung township in Myanmar. Medical examinations consisted of interviews regarding subjective neurological symptoms and objective neurological examinations of sensory disturbances. For subjective neurological symptoms, we ascertained the presence or absence of defects in smell, vision, taste, and hearing; the feeling of weakness; and chronic numbness or pain. For objective sensory disturbances, we examined defects in pain sensation, vibration sensation, and two-point discrimination. We analyzed the relationship between the subjective symptoms, objective sensory disturbances, and ACDW.

**Results:**

Residents with ACDW ≥ 10 parts per billion (ppb) had experienced a “feeling of weakness” and “chronic numbness or pain” significantly more often than those with ACDW < 10 ppb. Residents with ACDW ≥ 50 ppb had three types of sensory disturbances significantly more often than those with ACDW < 50 ppb. In children, there was no significant association between symptoms or signs and ACDW.

**Conclusion:**

Subjective symptoms, probably due to peripheral neuropathy, occurred at very low ACDW (around 10 ppb). Objective peripheral nerve disturbances of both small and large fibers occurred at low ACDW (> 50 ppb). These data suggest a threshold for the occurrence of peripheral neuropathy due to arsenic exposure, and indicate that the arsenic concentration in drinking water should be less than 10 ppb to ensure human health.

## Introduction

Heavy metals are naturally present in earth’s crust, but human activities have dramatically altered their geochemical cycles [1]. As the human population continues to increase, increasing numbers of people consume groundwater contaminated by arsenic [2]. At least 140 million people in more than 50 countries are exposed to arsenic-contaminated drinking water [3]. Chronic arsenic toxicity induces not only cardiovascular disease, skin lesions, diabetes, and cancers of the kidney, skin, and bladder [1, 4, 5] but also central and peripheral nervous system impairments [6–9].

In regard to the peripheral nervous system, a considerable number of patients who experience chronic arsenic exposure report subjective complaints of numbness or tingling [7, 10], but nerve conduction studies reveal no abnormal findings [11]. As for the central nervous system, the central sensory conduction time of patients with chronic arsenic exposure is longer than that in age-matched normal controls [6]. Neurobehavioral functions are impaired in patients aged 12–16 years who have experienced chronic arsenic exposure [8]. On the other hand, the latencies of brainstem auditory-evoked potentials do not differ between patients exposed to arsenic and normal controls [12].

**Fig. 1:**
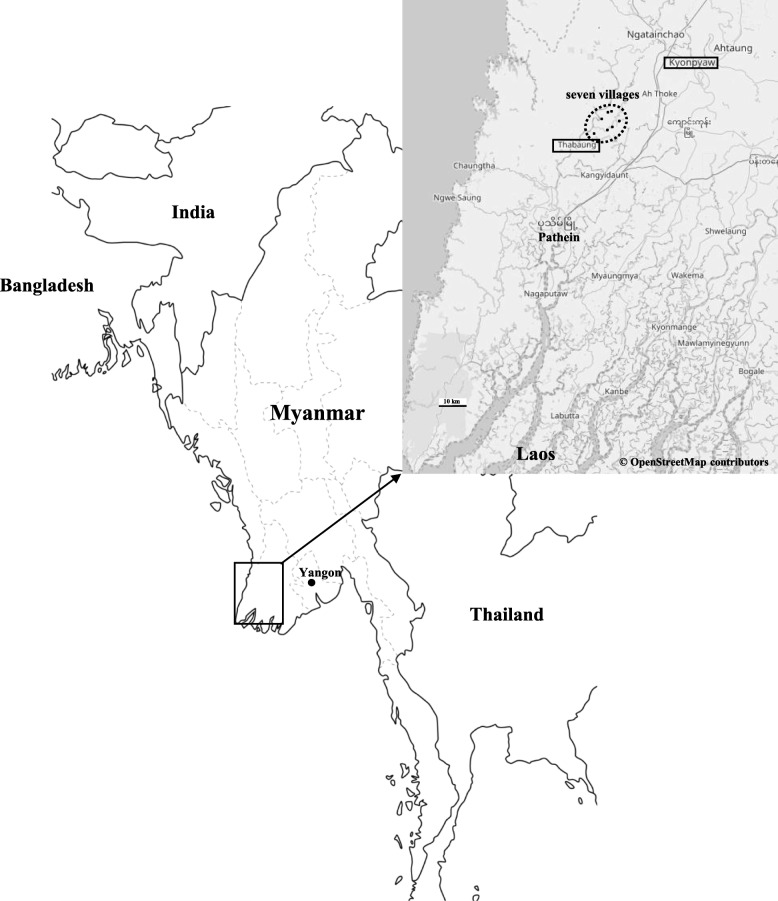
A map of Myanmar. The Thaboung and Kyonpyaw townships are indicated by square boxes. The location of the seven villages is indicated by an ellipse (dashed line)

Previous studies [13, 14] measured elevated arsenic concentrations in groundwater in the lower Ayeyawady basin in Myanmar. Prior to this research, in March 2015, we measured the heavy metal concentrations in the water from 23 tube wells in Kyonpyaw township, which is located in the same lower Ayeyawady basin as Thabaung township (Fig. 1) [15]. The average concentrations of six heavy metals (arsenic, cadmium, lead, chromium, copper, and nickel) for which the World Health Organization (WHO) sets reference values [16] were all below the reference values, except for arsenic (mean arsenic concentration = 64.7 parts per billion [ppb], WHO reference value = 10 ppb). Of these heavy metals, only arsenic contamination was assumed to be a problem for human health. We selected several villages in Thabaung township to investigate in this study because these villages did not have a transport network; therefore, the transport of both people and food were very limited. We measured arsenic concentrations in the tube well water in these villages and performed medical assessments of subjective neurological symptoms and objective neurological findings, focusing especially on sensory disturbances. In this study, we investigated whether neurological symptoms and findings were related to the arsenic concentration of drinking water (ACDW).

## Participants and methods

### Study design

A cross-sectional study design was employed. The study protocol was approved by the Institutional Review Board of the University of Miyazaki and was carried out in accordance with the Declaration of Helsinki [17]. Informed consent was obtained from all participants. If the participant was a child (under 8 years old), we obtained both oral informed consent from the subject and written informed consent from one of the parents.

### Participants

Medical examinations were performed from August to September 2016 in seven villages in the Thabaung township in Myanmar: Konetangyi, Shannkwin, Thayattaw, Yaylegyi, Latechaung, Dale-et, and Htanzinhla (Fig. 1). Residents in these inland villages eat rice, chicken, pork, river fish, and vegetables cultivated with rainwater. The seven villages do not have a transport network, and it takes 30–150 min to travel by boat to the city area (Fig. 1). The supply of electricity is very limited and there are few personal refrigerators. Because fresh food is uncommon, the residents rarely eat seafood. Many residents change their source of drinking water according to the season. For these residents, the source during the rainy season is river water or groundwater, whereas during the dry season it is only groundwater. We speculated that most arsenic exposure was due to groundwater. Medical examinations consisted of interviews regarding subjective neurological symptoms and neurological examinations of sensory disturbances. A total of 2081 participants were examined. Since examination findings of young children under 4 years of age were often not reproducible, we excluded these data from our analyses. The final number of participants was 1867.

### Subjective neurological symptoms

To obtain data on subjective neurological symptoms, face-to-face interviews of all participants were performed by our medical staff. Interviews were used to collect the following data: sex; age; presence or absence of defects in smell, vision, taste, or hearing; presence or absence of limb weakness; and presence or absence of chronic numbness or pain in the limbs.

### Objective sensory disturbances

We evaluated three types of sensory disturbances: pain (surface sensation), vibration (deep sensation), and two-point discrimination (combined sensation). Two Myanmarian neurologists, two Myanmarian physicians, and a Japanese neurologist with an interpreter performed all neurological examinations. A meeting was held in advance to unify neurological examination procedures and interpretations of neurological findings.

Pain sensation was evaluated on the dorsal side of the right hand and foot, relative to the skin on the sternum, using a sharp toothpick. The neurologist repeatedly touched the dorsum of the hand or foot and the skin of the sternum, applying equal pressure, and asked about the number of touches on the skin of the hand (or foot). In this test, 10 was considered normal and 0 indicated complete sensation loss. If the average value of the hand and foot was lower than 9, the subject’s pain sensation was judged to be impaired.

Vibration sensation was evaluated on the right ulnar head and lateral malleolus, compared with the sternum, using a 128-Hz tuning fork. The neurologist vibrated the tuning fork strongly and repeatedly touched the right ulnar head (or lateral malleolus) and sternum of the participant, and asked about the number of touches on the ulnar head (or lateral malleolus). Similar to the pain sensation test, 10 was considered normal and 0 indicated complete sensation loss. If the average value of the hand and foot was lower than 9, the subject’s vibration sensation was judged to be impaired.

Two-point discrimination thresholds were evaluated using a plastic aesthesiometer device, the Touch-Test Two-point Discriminator (Baseline, USA). The palmar surface of the right index finger tip was tested. Participants kept their eyes closed and were told that one or two ends of the aesthesiometer would touch their finger in a random sequence. In each trial, the neurologist applied equal pressure for 1 s. After detecting the touch, participants had to indicate the number of points they felt, i.e., one or two. For each response, the two-point distance was increased by 1 mm. The threshold was defined as the minimum two-point distance detected by participants in three consecutive trials: 4 mm or less was judged as normal, and 5 mm or more was judged as impaired.

### Arsenic concentration of drinking water

Between December 2015 and March 2016 (dry season), we used an inductively coupled plasma optical emission spectrometer (Optima 8000, Perkin Elmer, USA) to measure arsenic concentrations, including inorganic arsenic (III) and arsenic (V), in tube well water samples collected from the seven villages [18]. In total, arsenic concentrations were measured in 183 tube well water samples (< 50 ppb, 51 wells; 50–100 ppb, 91 wells; > 100, 41 wells). However, 796 of 1867 residents changed the source of their drinking water according to the season. For example, during the rainy season (5 months: June to October), residents drank river water (i.e., rainwater) (arsenic concentration = 0 ppb), but during the dry season (7 months: November to May) they drank water from tube wells (e.g., arsenic concentration = 80 ppb). In such cases, arsenic concentration in drinking water (ACDW) was calculated as (0 ppb * 5/12 year + 80 ppb * 7/12 year) = 46.7 ppb. In this study, we investigated whether two reference ACDW values of 10 ppb (very low concentration) and 50 ppb (low concentration) were associated with the presence of subjective neurological symptoms and/or objective sensory disturbances.

The ACDW reference value of 10 ppb was defined by the WHO as necessary to protect health [16, 19]. While some countries have officially adopted this value, many countries, including Myanmar, use a standard of 50 ppb, established in an earlier edition of the WHO guidelines [19, 20]. In this study, we divided residents into three groups according to their ACDW: very low (VLOW), < 10 ppb; low (LOW), 10–50 ppb; and moderate (MOD), ≥ 50 ppb. We then analyzed differences in the presence or absence of each symptom or sensory disturbance among the three groups.

### Statistical analyses

The three groups defined in the previous section were compared statistically using the chi-square and Ryan’s tests [21]. After these analyses, we performed additional analyses to determine the threshold of ACDW for neurological symptoms or findings. In these threshold analyses, participants were divided into two groups, ACDW < 10 ppb and ACDW ≥10 ppb, and the presence or absence of subjective neurological symptoms or objective sensory disturbances was analyzed by the chi-square test. In the same way, participants were divided into two groups, ACDW < 50 ppb and ACDW ≥ 50 ppb, and the presence of the same symptoms or findings was again analyzed by the chi-square test. In addition, to investigate the toxic effect of arsenic in children, the same analyses were performed in residents 5–15 years of age. *P* < 0.05 was considered statistically significant. The SPSS software version 22 was used for all statistical analyses.

## Results

### Subjective neurological symptoms

Data on subjective neurological symptoms were obtained from all participants above the age of 5 (*N* = 1867; male, *n* = 728; female, *n* = 1139; age [years, mean ± SD], 35.2 ± 20.4). Comparisons of subjective neurological symptoms among the VLOW, LOW, and MOD groups (chi-square test) are shown in Table 1. Residents in the VLOW group experienced the symptom “feeling of weakness” significantly less frequently than those in the LOW group (chi-square and Ryan test in Table 1). Subgroup analyses of children revealed no significant differences in any symptoms (*n* = 456; male, *n* = 223; female, *n* = 233; age [years, mean ± SD], 9.3 ± 3.0).

**Table 1:** Subjective neurological symptoms and arsenic concentration in drinking water (ACDW)

	Symptom	VLOW (< 10 ppb)	LOW (10–50 ppb)	MOD (≥ 50 ppb)	*P* (chi-square)
All participants (≥ 5 years old, *N* = 1867)					
Smell defects	Yes	13 (1.4)	11 (1.9)	3 (0.9)	0.446
	No	922	573	345	
Visual defects	Yes	298 (31.9)	188 (32.2)	100 (28.7)	0.616
	No	637	396	248	
Taste defects	Yes	12 (1.3)	11 (1.9)	8 (2.3)	0.401
	No	923	573	340	
Hearing defects	Yes	63 (6.7)	46 (7.8)	22 (6.4)	0.639
	No	872	540	324	
Feeling of weakness	Yes	122 (13.0)	110 (18.8)	54 (15.5)	0.020*^,a^
	No	813	474	294	
Chronic numbness or pain	Yes	191 (20.4)	151 (25.9)	79 (22.7)	0.095
	No	744	433	269	
Children (5–15 years old, *n* = 456)					
Smell defects	Yes	0 (0)	0 (0)	0 (0)	
	No	240	126	90	
Visual defects	Yes	8 (3.3)	4 (3.2)	1 (1.1)	0.549
	No	232	122	89	
Taste defects	Yes	0 (0)	0 (0)	0 (0)	
	No	240	126	90	
Hearing defects	Yes	2 (0.8)	1 (0.8)	2 (2.2)	0.523
	No	238	125	88	
Feeling of weakness	Yes	8 (3.3)	2 (1.6)	3 (3.3)	0.614
	No	232	124	87	
Chronic numbness or pain	Yes	9 (3.8)	2 (1.6)	5 (5.6)	0.295
	No	231	124	85	

Numbers indicate the number of participants

Numbers in parentheses indicate the percentage of “yes” participants in each ACDW group

Statistical analyses were performed using the chi-square test. *P < 0.05 indicates a statistically significant difference

Yes = present; no = absent

^a^VLOW < LOW (Ryan’s test, *P* < 0.05)

The results of additional chi-square tests to calculate the ACDW threshold for the occurrence of subjective neurological symptoms are provided in Table 2. Participants with ACDW ≥ 10 ppb experienced a “feeling of weakness” and “chronic numbness or pain” significantly more frequently than those with ACDW < 10 ppb (*P* < 0.05). When the reference value was set to 50 ppb, no significant differences were detected. Subgroup analyses of children revealed no significant differences in any symptoms.

**Table 2:** Additional analyses to calculate the ACDW threshold for the occurrence of subjective neurological symptoms

	Symptom	< 10 ppb	≥ 10 ppb	*P*	< 50 ppb	≥ 50 ppb	*P*
All participants (≥ 5 years old, *N* = 1867)							
Smell defects	Yes	13 (1.4)	14 (1.5)	0.497	24 (1.6)	3 (0.9)	0.230
	No	922	918		1495	345	
Visual defects	Yes	298 (31.9)	288 (30.9)	0.344	486 (32.0)	100 (28.7)	0.132
	No	637	644		1033	248	
Taste defects	Yes	12 (1.3)	19 (2.0)	0.137	23 (1.5)	8 (2.3)	0.207
	No	923	913		1496	340	
Hearing defects	Yes	63 (6.7)	68 (7.3)	0.351	109 (7.2)	22 (6.4)	0.334
	No	872	864		1410	324	
Feeling of weakness	Yes	122 (13.0)	164 (17.6)	0.004**	232 (15.3)	54 (15.5)	0.483
	No	813	768		1287	294	
Chronic numbness or pain	Yes	191 (20.4)	230 (24.7)	0.016*	342 (22.5)	79 (22.7)	0.495
	No	744	702		1177	269	
Children (5–15 years old, *n* = 456)							
Smell defects	Yes	0 (0)	0 (0)		0 (0)	0 (0)	
	No	240	216		366	90	
Visual defects	Yes	8 (3.3)	5 (2.3)	0.358	12 (3.3)	1 (1.1)	0.237
	No	232	211		354	89	
Taste defects	Yes	0 (0)	0 (0)		0 (0)	0 (0)	
	No	240	216		366	90	
Hearing defects	Yes	2 (0.8)	3 (1.4)	0.451	3 (0.8)	2 (2.2)	0.257
	No	238	213		363	88	
Feeling of weakness	Yes	8 (3.3)	5 (2.3)	0.358	10 (2.7)	3 (3.3)	0.490
	No	232	211		356	87	
Chronic numbness or pain	Yes	9 (3.8)	7 (3.2)	0.486	11 (3.0)	5 (5.6)	0.190
	No	231	209		355	85	

Numbers indicate the number of participants

Numbers in parentheses indicate the percentage of “yes” participants in each ACDW group

Statistical analyses were performed using the chi-square test. *P < 0.05, **P < 0.01, statistically significant difference

Yes = present; no = absent

### Objective sensory disturbances

Due to the lack of reproducibility of neurological findings related to sensory disturbances, reliable data were not obtained from 341 participants. Consequently, data on objective sensory disturbances were available for 1526 participants above the age of 5 (male, *n* = 567; female, *n* = 959; age [years, mean ± SD], 40.6 ± 17.5).

The results of chi-square tests to evaluate differences in objective sensory disturbances between the VLOW, LOW, and MOD groups are provided in Table 3. Participants in the VLOW and LOW groups experienced pain significantly less frequently than those in the MOD group, and those in the LOW group demonstrated impaired two-point discrimination less frequently than those in the MOD group (chi-square and Ryan’s tests in Table 3). Subgroup analyses of children revealed no significant differences in any sensory disturbances (*n* = 133; male, *n* = 68; female, *n* = 65; age [years, mean ± SD], 11.7 ± 2.6).

**Table 3:** Objective sensory disturbances and arsenic concentration of drinking water (ACDW)

	Impairment	VLOW (< 10 ppb)	LOW (10–50 ppb)	MOD (≥ 50 ppb)	*P* (chi-square)
All participants (> 5 years old, *n* = 1526)					
Pain sensation	Yes	38 (5.0)	21 (4.3)	25 (9.0)	0.021*^,a^
	No	726	463	253	
Vibration sensation	Yes	33 (4.3)	14 (2.9)	18 (6.5)	0.070
	No	731	470	260	
Two-point discrimination	Yes	34 (4.5)	11 (2.3)	20 (7.2)	0.006*^,b^
	No	730	473	258	
Children (5–15 years old, *n* = 133)					
Pain sensation	Yes	3 (3.8)	0 (0)	2 (9.1)	0.243
	No	77	31	20	
Vibration sensation	Yes	1 (1.3)	0 (0)	2 (9.1)	0.060
	No	79	31	20	
Two-point discrimination	Yes	2 (2.5)	0 (0)	0 (0)	0.516
	No	78	31	22	

Numbers indicate the number of participants

Numbers in parentheses indicate the percentage of “yes” participants in each ACDW group

Statistical analyses were performed using the chi-square test. *P < 0.05; statistically significant difference

Yes = present; no = absent

^a^VLOW and LOW < MOD

^b^LOW < MOD (Ryan’s test, *P* < 0.05)

The results of additional chi-square tests to calculate the ACDW threshold for the occurrence of objective sensory disturbances are provided in Table 4. Participants with ACDW ≥ 50 ppb experienced three types of sensory disturbances significantly more frequently than those with ACDW < 50 ppb (*P* < 0.05). When the reference value was set to 10 ppb, no significant differences were detected. Subgroup analyses of children revealed no significant differences in any sensory disturbances.

**Table 4:** Additional analyses for the ACDW threshold for the occurrence of objective sensory disturbances

	Impairment	< 10 ppb	≥10 ppb	*P*	< 50 ppb	≥ 50 ppb	*P*
All participants (> 5 years old, *n* = 1526)							
Pain sensation	Yes	38 (5.0)	46 (6.0)	0.212	59 (4.7)	25 (9.0)	0.005**
	No	726	716		1189	253	
Vibration sensation	Yes	33 (4.3)	32 (4.2)	0.504	47 (3.8)	18 (6.5)	0.036*
	No	731	730		1201	260	
Two-point discrimination	Yes	34 (4.5)	31 (4.1)	0.800	45 (3.6)	20 (7.2)	0.007**
	No	730	731		1203	258	
Children (5–15 years old, *n* = 133)							
Pain sensation	Yes	3 (3.8)	2 (3.8)	0.664	3 (2.7)	2 (9.1)	0.191
	No	77	51		108	20	
Vibration sensation	Yes	1 (1.3)	2 (3.8)	0.349	1 (0.9)	2 (9.1)	0.071
	No	79	51		110	20	
Two-point discrimination	Yes	2 (2.5)	0 (0)	0.360	2 (1.8)	0 (0)	0.695
	No	78	53		109	22	

Numbers indicate the number of participants

Numbers in parentheses indicate the percentage of “yes” participants in each ACDW group

Statistical analyses were performed using the chi-square test. *P < 0.05, **P < 0.01, statistically significant difference

Yes = present; no = absent

## Discussion

### Subjective neurological symptoms

Comparisons among the three groups indicated that the participants in the VLOW group reported a “feeling of weakness” significantly less frequently than those in the LOW group (Table 1). In the additional analyses (Table 2), participants with ACDW ≥ 10 ppb were more likely to report a “feeling of weakness” than those with ACDW < 10 ppb (Table 2). Therefore, the ACDW threshold for the symptom “feeling of weakness” was estimated to be about 10 ppb. On the other hand, comparisons of “chronic numbness or pain” among the three groups revealed no significant differences. In the additional analyses (Table 2), participants with ACDW ≥ 10 ppb were more likely to report “chronic numbness or pain” than those with ACDW < 10 ppb. Therefore, the ACDW threshold for the symptom “chronic numbness or pain” was estimated to lie between 10 and 50 ppb. While this range is quite low in absolute terms, an arsenic concentration of 50 ppb in drinking water, which is the Myanmar reference standard, may cause some neurological impairments.

Several studies have shown that arsenic exposure induces peripheral neuropathy or neuritis [7, 9, 22–24]. Two subjective motor and sensory symptoms in this study, a “feeling of weakness” and “chronic numbness or pain,” were significantly less frequent in participants with very low or low ACDW, and might be related to peripheral neuropathy.

Because only a small amount of arsenate can penetrate the central nervous system (CNS) via the blood-brain barrier [25, 26], CNS impairment may not be easily induced by low ACDW (50 ppb). Therefore, low ACDW is not predicted to significantly influence the four subjective symptoms we examined (i.e., defects in smell, vision, taste, and hearing).

### Objective sensory disturbances

Pain and vibration sensations represent the function of small and large peripheral nerve fibers, respectively. In this study, participants with ACDW ≥ 50 ppb were more likely to report disturbances of pain and vibration than those with ACDW < 50 ppb, although there was no significant difference in vibration disturbance among the three groups (Tables 3 and 4). The thresholds for pain and vibration sensory disturbances were both estimated to be about 50 ppb. In other words, objective peripheral neuropathies in small and large nerve fibers are induced by low ACDW (50 ppb).

As mentioned above, several studies have shown that arsenic exposure induces peripheral neuropathy or neuritis [7, 9, 22–24]. A few studies have separately analyzed the function of small and large fibers, and suggested that these particular effects of arsenic are specific to small fibers [6, 9, 10]. Large-fiber function can be evaluated not only by neurological examination of vibration sensation but also by nerve conduction studies. A study performed in Inner Mongolia reported that chronic arsenic exposure from drinking water (mean arsenic level in tube well, 158.3 ppb; total number of participants, 134) did not affect nerve conduction velocity [11]. In this study, for the first time, we analyzed nerve function according to nerve diameter (small and large fibers) and found that both small and large peripheral nerve fibers were impaired by low ACDW (50 ppb).

Participants with ACDW ≥ 50 ppb had more disturbances in two-point discrimination than those with ACDW < 50 ppb. The two-point discrimination method reflects combined sensation, involving the peripheral and central nervous systems [27]. A previous report speculated that the sensory pathway of the central nervous system is impaired by high-dose arsenic exposure [6]. However, the observed disturbances in two-point discrimination likely reflected impairments of peripheral nerves, although it is impossible to rule out the possibility of central nervous system impairment by low ACDW.

### Exposure–response relationship

Exposure dose–response relationships of arsenic have been reported in previous studies [28]. An increased prevalence of skin lesions was observed even at an ACDW level of 5–10 ppb [5, 28]. In an analysis of internal malignancies and arsenic exposure, the dose–response relationships for the occurrence of lung, bladder, and kidney cancers were linear [28, 29]. In a survey of 1185 people in the USA, those who consumed ACDW ≥ 10 ppb were statistically more likely to report a history of circulatory problems [30]. A significant dose–response relationship was also observed between arsenic exposure and serum hepatic enzyme levels, with statistically higher levels found in subjects who consumed ACDW ≥ 34 ppb [31]. In the context of neurological impairments, arsenic exposure–response relationships have rarely been reported. Here, based on our findings, subjective neurological impairment occurred at an ACDW level of 10 ppb, and objective peripheral nerve disturbances occurred at ACDW ≥ 50 ppb.

### Effect of low ACDW on children

It remains controversial whether low ACDW is likely to affect the intellectual functions of children [32, 33]. An epidemiological study indicated that CNS impairments such as neurocognitive or intellectual deficits were associated with arsenic exposure in children [33, 34]. A study in West Bengal, however, showed no associations between long-term arsenic exposure in water and intellectual functions in children [32]. In this study, ACDW was not significantly associated with neurological symptoms or sensory disturbances in children. We consider three possible explanations for this observation. First, exposure durations in children are shorter than those in adults. In fact, for all subjective symptoms and neurological findings, the incidence of abnormalities increased with age (*P* < 0.05, Spearman rank correlation test, data not shown). However, exposure duration was difficult to accurately evaluate because no data were available regarding arsenic concentrations in tube wells until several years ago. Second, children may have a higher arsenic methylation capacity than adults [35, 36], resulting in more efficient detoxification [36] and a lower incidence of neuropathy. The third possible explanation is essentially a statistical artifact: the number of children in this study was quite small (subjective neurological symptoms, 456; objective sensory disturbances, 133).

### Limitations

While this study has the advantage of surveying a large number of residents in area with little transport network, it has four limitations. First, it used a cross-sectional design. Average ACDW was used to represent arsenic exposure, but the cumulative amount of arsenic intake over one’s lifetime might also be important. Because no data are available regarding the arsenic concentration in tube wells prior to several years ago, we cannot be sure that the current ACDW is the same as it was in the past. Therefore, we did not attempt to analyze cumulative arsenic levels. Second, arsenic exposure may differ between the rainy and dry seasons (June to October and November to May, respectively). Specifically, arsenic exposure in the rainy season is lower because residents frequently drink river water (arsenic concentration = 0 ppb) during this period. We performed neurological examinations during the rainy season (from August to September 2016). We do not know how long neurological impairment persists, but the discrepancy in arsenic concentration between the rainy and dry seasons may have affected our findings regarding neurological impairments. Third, the measurement technique used in this study, inductively coupled plasma optical emission spectrometry, measured inorganic arsenic (III) and arsenic (V) in drinking water [37]. Although organic arsenic is less toxic than inorganic arsenic [35, 37], we did not measure organic arsenic in this study, and we cannot completely exclude the possibility that organic arsenic affected the results. Fourth, the arsenic concentration in each tube well was measured only once in the dry season. Although two studies showed that seasonal variations had no clear effect on groundwater arsenic concentrations [38, 39], a study in India suggested that a maximum change of 30% may occur [40]. Thus, the ACDW measured in the dry season in this study may have differed slightly from the true value.

## Conclusions

Subjective symptoms, such as a feeling of weakness and chronic numbness or pain, occurred at very low ACDW (around 10 ppb). Objective peripheral nerve disturbances of both the small- and large-fiber types occurred at low ACDW (more than 50 ppb). These data suggest a possible threshold for the occurrence of peripheral neuropathy due to arsenic exposure. Based on our findings, ACDW ≥ 10 ppb may cause some neurological impairments, implying that an ACDW level of 10 ppb, based on the WHO guideline [19, 41], is an appropriate threshold. We did not obtain any evidence that arsenic exposure is likely to exert a particular effect on the peripheral or central nervous system in children.

## Abbreviation

ACDW: Arsenic concentration in drinking water
